# Infections Deaths in the PLATO Trial

**DOI:** 10.1055/s-0041-1736638

**Published:** 2021-11-08

**Authors:** Victor Serebruany, Jean-Francois Tanguay

**Affiliations:** 1 Department of Neurology, Johns Hopkins University, Baltimore, Maryland, United States; 2 Interventional Cardiology, Montreal Heart Institute, Université de Montréal, Montreal, Quebec, Canada

**Keywords:** clinical trial, ticagrelor, clopidogrel, death, infections, pneumonia, sepsis

## Abstract

**Background** Cardiovascular benefits of aggressive dual antiplatelet therapy may be associated with extra risks including bleeding, cancer, and infections discovered first for prasugrel in the TRial to assess Improvement in Therapeutic Outcomes by optimizing platelet InhibitioN with prasugrel (TRITON) trial. Ticagrelor in PLATO also caused slightly more infections but surprisingly less sepsis-related deaths (SRD) than clopidogrel. However, verified infection fatalities in PLATO were lacking from the public domain. We obtained the complete Food and Drug Administration (FDA)-issued primary causes death list, matched it with the few local site records dataset and analyzed the patterns of infections and deaths reported in PLATO.

**Methods** Among infections, the FDA spreadsheet contains only two primary death codes for pneumonia (12–2) and SRD (12–8). We obtained local evidence for two pneumonia and two SRD and matched those with the FDA records. We assessed how SRD patterns were reported among nonvascular death's dataset.

**Results** The FDA PLATO records indicate that clopidogrel caused numerically less ( *n* = 8) primary pneumonia deaths than ticagrelor ( *n* = 10) but over three times more SRD ( *n* = 23/7). Among matched verifiable outcomes, both pneumonia deaths were correct, but two clopidogrel SRD were incorrect. Of the remaining 21 clopidogrel SRD, 6 were reported as two separate closed paired entries in Brazil (lines 76 and 78 and 86 and 88) and India (lines 436 and 440), suggesting last minute addition of potentially incorrect SRD reports. Four ticagrelor SRD (lines 24,193,467 and 650) were “compensated” with close or next in line clopidogrel SRD entries (lines 22,195,468 and 651).

**Conclusion** The FDA-issued evidence suggests no benefit of ticagrelor in preventing deaths from infections with slightly more pneumonia deaths, with possible misreporting of SRD in PLATO. These findings require an in-depth precise review of sepsis deaths in this trial.

## Introduction

The relations between infections, hemostasis, and potency of antithrombotic therapy are intertwined but important, especially after utilization of current aggressive dual antiplatelet strategies following coronary revascularization. 1 Indeed, such complex interventions per se often require use of numerous devices into and out of the arterial circulation, and these procedures may cause bacteremia 2 or even septicemia. 3 Since already established shortcomings following clopidogrel may include impaired wound healing and increased postsurgery infections, 4 5 more powerful antiplatelet strategies could present even greater risks. The mechanism responsible for such harmful association is probably indirect and involves weakening of platelet–neutrophil–endothelial cross-talk necessary to combat infections, and/or keep inflammation from spreading. However, the comparative risks of infections including sepsis among adverse events in such patients have not been identified. The first alarming signal that potent long-term antiplatelet therapy may cause excess of infections that was observed in the prasugrel arm of TRITON-TIMI 38 trial. 6 Consistently, ticagrelor in PLATO caused more infections but surprisingly less sepsis-related deaths (SRD) than clopidogrel. 7 The details of the Food and Drug Administration (FDA) review 7 are outlined in Table 1 .

**Table 1:** FDA analyses of infections and sepsis-related deaths in PLATO

Infection	Ticagrelor	Clopidogrel
Upper respiratory	947 (10.25%)	882 (9.6%)
Lungs	233 (2.52%)	245 (2.67%)
Urinary tract	184 (2.0%)	161 (1.8%)
Viral	466 (5.05%)	415 (4.52%)
Bacterial	506 (5.48%)	492 (5.36%)
Any infection	1,488 (16.11%)	1,438 (15.65%)
Fever	331 (3.58%)	318 (3.46%)
Sepsis-related deaths	7 (0.1%)	23 (0.2%)

Abbreviation: FDA, Food and Drug Administration.

The data outlined in the Table 1 strongly suggest that more profound platelet inhibition with ticagrelor causes a slightly greater risk for infections than after clopidogrel. Such observation may be related to the fact that ticagrelor PLATO regiment was more potent. However, how could the reduction of SRD reported after ticagrelor therapy be reconciled? With details unavailable for public these numbers should be independently verified, despite some preliminary attempts to explain this paradox. 8 9 10 We recently gained access to the detailed FDA-issued dataset of 938 PLATO deaths which has been matched with local patient-level data from sites controlled by the sponsor revealing that actual existence, the precise dates, and the proper causes of some deaths in PLATO were inaccurately reported in favor of ticagrelor. 11 Moreover, there is a massive discrepancy between primary death causes reported to the FDA, and those utilized by the PLATO Investigators for numerous secondary overoptimistic reports published in top journals for over a decade. 12 Examining cancer deaths reveled that many clopidogrel events were misreported in PLATO favoring ticagrelor as well. 13 Here, we disclose verified deaths from pneumonia and sepsis in PLATO, examining their reporting patterns and validity.

## Methods

Based on the Freedom of Information Act, BuzzFeed filed a legal complaint in U.S. Federal Court, won an expedited order, and shared with us the complete PLATO death list submitted to the FDA by the ticagrelor sponsor. The FDA spreadsheet contains 938 PLATO deaths with trial identification numbers, country, enrolling site, patient age, gender, treatment assignments, discontinuations, outcome codes, dates, and precise causes of trial exit. Each event contains whether the death cause was vascular (code 11), nonvascular (code 12), or unknown (code 97). There were 14 subcodes for vascular, 9 subcodes for nonvascular deaths, and universal code “99” which applied for “other” causes. Among infections, the spreadsheet contains primary deaths' codes for pneumonia (12–2) and SRD (12–8) only. Most of the data were controlled and reported by PLATO sponsor, with the exception of the United States, Russia, Georgia, and most (sites 5101–5106) of Ukraine. The entire United States was monitored by ReSearch Pharmaceutical Services, (Wort Washington, Pennsylvania, United States; *http://www.rpsweb.com* ). All Russian, Georgian, and most Ukrainian sites were monitored by Evidence CRP, now Worldwide Clinical Trials, (Morrisville, North Carolina, United States; *http://wwctrials.com/* ). The FDA-issued list contains 18 precisely detailed pneumonia deaths and 30 SRD. We have local verified records on four of such deaths (two each for pneumonia and SRD) among 861 PLATO patients from 14 enrolling sites in eight countries and matched those with what was reported to the FDA. We also assessed the reporting pattern of deaths from infections issued by the FDA just scrolling down column “S” for the nonvascular death causes.

## Results

Among 18 FDA-reported pneumonia deaths in PLATO, those attributed to ticagrelor ( *n* = 10) were numerically more than after clopidogrel ( *n* = 8). We matched two PLATO patients with the local site data (one ticagrelor and one clopidogrel). Both cases were reported correctly. With regard to SRD verification in two clopidogrel cases, both primary death causes were reported incorrectly. As reported by site the primary cause of death of one clopidogrel patient was multiorgan failure (nonvascular subcode 9) but not SRD (subcode 8). Another patient is of significant interest since sepsis was among the secondary diagnoses. However, site reported respiratory failure (nonvascular subcode 1) as a primary death cause but not sepsis. Of the remaining 21 clopidogrel SRD, 6 were reported as three separate pairs repeating previous in list patient record suggesting last minute addition of incorrect cases. In contrast, four ticagrelor SRD has been accompanied by very close clopidogrel SRD entry in a pattern to “compensate” or maintain ticagrelor sepsis advantage. See Table 2 for details.

**Table 2:** FDA-issued dataset entries for questionable sepsis deaths in PLATO trial

ENTR	Country	Age	ETN	Gender	STUDYDY	TRTRTXT	NVASSCLS
22	Argentina	57	E1016xxx2DE	Female	37	Clopidogrel	8
24	Argentina	71	E1016xxx4DE	Male	8	Ticagrelor	8
76	Brazil	59	E1422xxx1DE	Male	52	Clopidogrel	8
78	Brazil	77	E1425xxx11DE	Female	147	Clopidogrel	8
86	Brazil	64	E1427xxx6DE	Female	56	Clopidogrel	8
88	Brazil	75	E1427xxx5DE	Male	34	Clopidogrel	8
193	Czech	67	E1804xxx9DE	Female	69	Ticagrelor	8
195	Czech	62	E1805xxx4DE	Female	51	Clopidogrel	8
436	India	74	E2717xxx37DE	Female	198	Clopidogrel	8
440	India	74	E2719xxx8DE	Female	83	Clopidogrel	8
467	Indonesia	52	E2805xxx1DE	Female	131	Ticagrelor	8
468	Israel	70	E2901xxx79DE	Female	169	Clopidogrel	8
650	Poland	73	E3625xxx45DE	Female	242	Ticagrelor	8
651	Poland	71	E3625xxx72DE	Female	17	Clopidogrel	8

Abbreviations: ENTR, patient number among 938 reported PLATO deaths, goes in alphabetical order from Argentina to the United States, ending with 2 last deaths from Ukraine after monitoring switch from CRO to the sponsor; ETN, event tracking number; FDA, Food and Drug Administration; STUDYDY, Study days; TRTRTXT, randomization treatment text; NVASCLS, subclassification of nonvascular death code. 12

The surprising and highly unusual pattern of three pairs of close or next in line clopidogrel patients marked as SRD can be easily detected by just scrolling down Excel list column “S” among fatalities in Brazil and India. Repeated placement in pairs of subcode 8 (SRD) for 6 clopidogrel patients could indicate database manipulation or/and last-minute modifications to artificially worsen clopidogrel infection risks. Interestingly, patients in between: on line 77 received ticagrelor but patient on line 87 was on clopidogrel. However, that particular patient reported on line 87 experienced a cardiogenic shock, the “precious” vascular cause of death potentially contributing to PLATO primary efficacy outcome, and next in line 88 deceased clopidogrel patient was reported as SRD.

## Impression

The main finding of this report suggests that ticagrelor is not better than clopidogrel with regard to risks of infections and affiliated deaths. Aside from possible misreporting, and unsubstantiated claims that ticagrelor could prevent SRD in PLATO, the drug per se probably do not cause direct inflammation effect but could negatively contribute via excessive chronic platelet inhibition when used in full-dose long term. Assessing infections signal after ticagrelor was tricky because of more infections, but over three times, less SRD than after clopidogrel were reported in PLATO. In contrast to the balanced and mildly concerned FDA report, 7 the secondary PLATO publications overoptimistically present the infections data as somewhat a protective effect of ticagrelor. 8 9 10 14 15 16 Aside from reduction in ischemic cardiovascular events, the explanations for the mortality benefits of ticagrelor by suggesting pleiotropic effects 17 18 19 cannot be sustained since PLATO deaths benefit has been never achieved in later ticagrelor trials, making any extravagant explanation(s) meritless.

Long-term dual antiplatelet therapy may be associated with the unexpected but fatal complications including bleeding, infections, and SRD. This is especially alarming since modern antiplatelet strategies are often used off-label with regard to treatment duration. Also, randomized evidence suggests that most vascular benefits emerge early after coronary stenting but most complications including bleeding or/and infections grow over time of exposure. Unfortunately, we will not be able to intelligently assess the real rates of infections after dual antiplatelet therapy since the trials design do not measure such adverse events. However, more recent trials suggested that shorter antiplatelet strategies decrease bleeding risks without increased mortality.

The unremarkable and probably correct reporting of pneumonia deaths but possible increase of clopidogrel SRD count in PLATO was no surprise to the Task Force since changes of death dates, and especially their causes were already well-documented and previously reported. 11 What is puzzling are the observation of three pairs of clopidogrel SRD in Brazil and India justifying complete reassessment of PLATO deaths. Such misreporting of data or error in late process of submission could not be detected by the independent researchers or scientific executive committee. Furthermore, the FDA could pick-up such evidence without an independent new monitoring of all deaths in PLATO. The observation that such rare fatal outcomes as SRD are reported in pairs is highly questionable. Indeed, SRD were reported as a primary cause of death in less than 3.2% PLATO fatalities making these 3 pairs of clopidogrel deaths unusual and very unlikely. Such particular pattern of death reporting is very similar to cancer misreporting in PLATO when clopidogrel deaths were also entered in pairs. 12 There are few shortcomings of the present analyses limiting our abilities to draw definite conclusions. In fact, within any large-scale world-wide clinical trial to access the difference of 8 versus 10 fatal pneumonia events is challenging, and may represent a play of chance. The numbers are simply way too small to make any qualifying statements. The SRD misreporting will not necessarily change the direction of PLATO trial results evaluation since numerous issues gave been already reported. 11 12 13 Together with the FDA, we are currently implementing the joint status report in civil case number 21–572 based on the Freedom of Information Act in Washington, District of Columbia, District Court focusing on PLATO late event adjudication and some submission NDA 022433 evidence.

## Conclusion

The pneumonia deaths were reported correctly, while many SRD were misreported in PLATO favoring ticagrelor.
